# Recent Advances of Malaria Parasites Detection Systems Based on Mathematical Morphology

**DOI:** 10.3390/s18020513

**Published:** 2018-02-08

**Authors:** Andrea Loddo, Cecilia Di Ruberto, Michel Kocher

**Affiliations:** 1 Department of Mathematics and Computer Science, University of Cagliari, 09124 Cagliari, Italy; dirubert@unica.it; 2 Biomedical Imaging Group, École Polytechnique Fédérale de Lausanne (EPFL), 1015 Lausanne, Switzerland; michel.kocher@heig-vd.ch

**Keywords:** malaria, red blood cells segmentation, mathematical morphology, medical image analysis

## Abstract

Malaria is an epidemic health disease and a rapid, accurate diagnosis is necessary for proper intervention. Generally, pathologists visually examine blood stained slides for malaria diagnosis. Nevertheless, this kind of visual inspection is subjective, error-prone and time-consuming. In order to overcome the issues, numerous methods of automatic malaria diagnosis have been proposed so far. In particular, many researchers have used mathematical morphology as a powerful tool for computer aided malaria detection and classification. Mathematical morphology is not only a theory for the analysis of spatial structures, but also a very powerful technique widely used for image processing purposes and employed successfully in biomedical image analysis, especially in preprocessing and segmentation tasks. Microscopic image analysis and particularly malaria detection and classification can greatly benefit from the use of morphological operators. The aim of this paper is to present a review of recent mathematical morphology based methods for malaria parasite detection and identification in stained blood smears images.

## 1. Introduction

Haematology is the branch of medicine concerned with the study, diagnosis, monitoring, treatment, and prevention of diseases related to the blood and blood-forming organs. Haematology studies the blood in health and pathological conditions, firstly to identify the patient’s health condition and, secondly, to predict how the bone marrow may have contributed to reach that condition.

**Figure 1:**
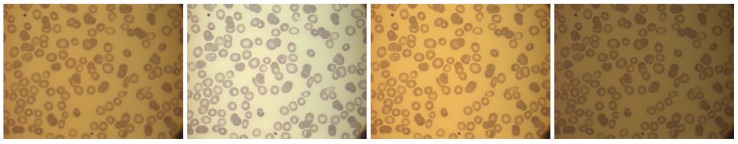
Different illumination conditions generate different images because of the absence of a standardized acquisition procedure. From left to right: acquisition of the same smear with four microscope’s brightness levels. Courtesy of CHUV, Lausanne.

Thus, haematology studies the relationship between the bone marrow and the systemic circulation. In fact, there are many diseases, disorders, and deficiencies that can affect the number and type of produced blood cells, their function and their lifespan. Usually, only normal, mature or nearly mature cells are released into the bloodstream, but certain circumstances can induce the bone marrow to release immature and/or abnormal cells into the circulation. One of the most frequently ordered tests to monitor the proportion of the cell components into the blood stream is the Complete Blood Count (CBC), which offers various haematologic data represented by the numbers and types of cells in the peripheral circulation. The cells percentage is compared with the reference ranges in order to determine if the cells are present in their expected percentage, if one cell type is increased, decreased or if immature cells exist. Reference ranges for blood tests are sets of values used to interpret a set of diagnostic test results from blood samples. Since it is difficult to prove that healthy-considered subjects may not have infections, parasitic infection and nutritional deficiency, it is more feasible to talk about reference ranges rather than normal ranges. A reference range is usually defined as the set of values in which 95% of the normal population falls within. It is determined by collecting data from vast numbers of laboratory tests result from a large number of subjects who are assumed to be representative of the population. With automatic counters or the flow cytometry, an automated CBC can be performed quickly. However, if the results from an automated cell count indicate the presence of abnormal cells or if there is a reason to suspect that abnormal cells are present, then a blood smear will be collected [1]. A blood smear is often used to categorize and/or identify conditions that affect one or more types of blood cells and to monitor individuals undergoing treatment for these conditions. The results of a blood smear typically include a description of the cells appearance, as well as any abnormalities that may be seen on the slide. The manual analysis of blood smears is tedious, lengthy, repetitive and it suffers from the presence of a non-standard precision because it depends on the operator’s skill. The use of image processing techniques can help to analyse, count the cells in human blood and, at the same time, to provide useful and precise information about cells morphology. Peripheral blood smears analysis is a common and economical diagnosis technique by which expert pathologists may obtain health information about the patients. Although this procedure requires highly trained experts, it is error-prone and could be affected by inter-observer variations. Moreover, blood cells’ images taken from a microscope could vary in their illumination and colouration conditions, as shown in Figure 1. Typical blood cells’ images contain three main components of interest: the platelets (or thrombocytes), the red blood cells (or erythrocytes) and the white blood cells (or leukocytes). It is worth considering that blood cells exist with different shapes, characteristics and colourations, according to their types. Many tests are designed to determine the number of erythrocytes and leukocytes in the blood, together with the volume, sedimentation rate, and haemoglobin concentration of the red blood cells (blood count). In addition, certain tests are used to classify blood according to specific red blood cell antigens, or blood groups. Other tests elucidate the shape and structural details of blood cells and haemoglobin and other blood proteins. Blood can be analysed to determine the activity of various enzymes, or protein catalysts, that either are associated with the blood cells or are found free in the blood plasma. Blood also may be analysed on the basis of properties such as total volume, circulation time, viscosity, clotting time and clotting abnormalities, acidity (pH), levels of oxygen and carbon dioxide, and the clearance rate of various substances. There are special tests based on the presence in the blood of substances characteristic of specific infections, such as the serological tests for syphilis, hepatitis, and human immunodeficiency virus (HIV, the AIDS virus) (https://www.britannica.com/topic/blood-analysis). Among the several available blood tests, the most common are certainly the blood cells counts, e.g., a CBC is a measure of the haematologic parameters of the blood. Included in the CBC is the calculation of the number of red blood cells (red blood cell count) or white blood cells (white blood cell count) in a cubic millimetre (mm^3^) of blood, a differential white blood cell count, a haemoglobin assay, a hematocrit, calculations of red cell volume, and a platelet count. The differential white blood cell count includes measurements of the different types of white blood cells that constitute the total white blood cell count: the band neutrophils, segmented neutrophils, lymphocytes, monocytes, eosinophils, and basophils. A specific infection can be suspected on the basis of the type of leukocyte that has an abnormal value [2].

**Figure 2:**
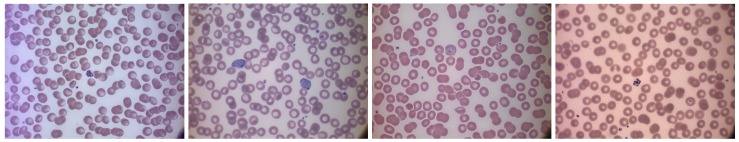
Types of human malaria parasites: from left to right, *P. falciparum* in its schizont stage, *P. vivax* in two gametocytes specimens and one ring stage, *P. ovale* in its ring stage, *P. malariae* in its schizont stage. Courtesy of CHUV, Lausanne.

**Figure 3:**
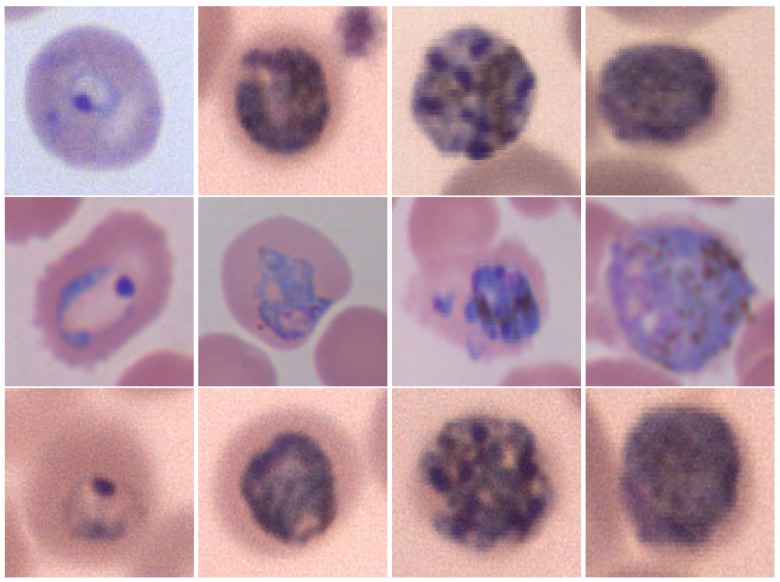

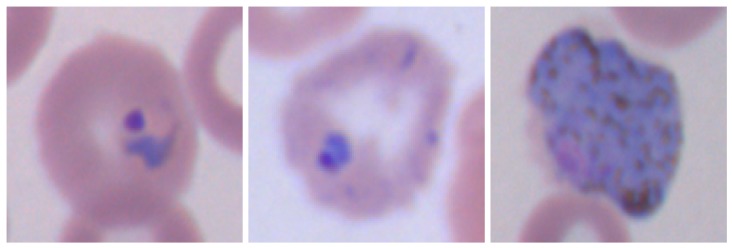
Examples of malaria parasite stages. First row, from left to right: *P. falciparum* ring, trophozoite, schizont, gametocyte; second row, from left to right: *P. ovale* ring, trophozoite, schizont, gametocyte; third row, from left to right: *P. malariae* ring, trophozoite, schizont, gametocyte; last row, from left to right: *P. vivax* ring, developed trophozoite, gametocyte. Courtesy of CHUV, Lausanne.

Human malaria infection is not strongly related to cell count, but it needs different tests in order to be identified. It can only be caused by parasitic protozoans belonging to the *Plasmodium* type. The parasites are spread to people through the bites of infected female Anopheles mosquitoes, called “malaria vectors”. There are five parasite species that cause malaria in humans and two of these species, *Plasmodium falciparum* and *Plasmodium vivax*, constitute the greatest threat. *Plasmodium ovale*, *Plasmodium malariae* and *Plasmodium knowlesi* are the three remaining species that are less dangerous in humans [3], as shown in Figure 2. All five species may appear in four different life-cycle stages during the infection phase in peripheral blood: ring, trophozoite, schizont and gametocyte. Some examples are shown in Figure 3. The life-cycle-stage of the parasite is defined by its morphology, size and the presence or absence of malarial pigment. The species differ in the changes of infected cell’s shape, presence of some characteristic dots and the morphology of the parasite in some of the life-cycle-stages [4].

Computer vision techniques for malaria diagnosis and recognition represent a relatively new area for early malaria detection and, in general, for medical imaging, able to overcome the problems related to manual analysis, which is performed by human visual examination of blood smears. The whole process requires an ability to differentiate between non-parasitic stained components/bodies (e.g., red blood cells, white blood cells, platelets, and artefacts) and the malarial parasites using visual information. If the blood sample is diagnosed as positive (i.e., parasites present), an additional capability of differentiating species and life-stages (i.e., identification) is required to specify the infection. Numerous methods of automatic malaria diagnosis have been proposed so far, in order to overcome the issues before mentioned. The aim of this paper is to review and analyse the works of different researchers who in particular have used mathematical morphology as a powerful tool for computer aided malaria detection and classification.

### Mathematical Morphology

Mathematical morphology (MM) can be defined as a theory for the analysis of spatial structures. It is called morphology because it aims at analysing the shape and form of objects. It is mathematical in the sense that the analysis is based on set theory, integral geometry, and lattice algebra. MM is not only a theory, but also a very powerful image analysis technique [5]. It was introduced by Matheron in 1964 as a technique for analysing geometric structure of metallic and geologic samples. It refers to a branch of nonlinear image processing and analysis that concentrates on the geometric structure within an image. The morphological filters, which can be constructed on the basis of the underlying morphological operations, are more suitable for shape analysis than the standard linear filters since the latter sometimes distort the underlying geometric form of the image. Some of the salient points regarding the morphological approach are as follows [6]:

- Morphological operations provide for the systematic alteration of the geometric content of an image while maintaining the stability of the important geometric characteristics.
- There exists a well-developed morphological algebra that can be employed for representation and optimization.
- It is possible to express digital algorithms in terms of a very small class of primitive morphological operations.
- There exist rigorous representation theorems by means of which one can obtain the expression of morphological filters in terms of the primitive morphological operations.

MM was initially developed for binary images and later on generalized to grayscale images [5,7], considered as a sampled function of $R^{2}$ in R, or in general of any function of $R^{n}$ in R. More recently, several researchers have extended morphological operators to colour (or in general multispectral) images, considered as sampled functions of $R^{n}$ in $R^{m}$, with *m* being equal to three in the case of the usual colour images or to the number of bands otherwise [8]. Moreover, several approaches for fuzzifying MM have been proposed, extending the ordinary morphological operations by using fuzzy sets [9].

Dilation and erosion are the basic morphological processing operations. They are defined in terms of more elementary set operations, but are employed as the basic elements of many algorithms. Both dilation and erosion are produced by the interaction of a set called structuring element (SE) with a set of pixels of interest in the image. The structuring element has both a shape and an origin. From these two basic operators, others have been derived (opening, closing, hit-or-miss). They can be applied to extract image components useful in the representation and descriptions of region shapes, such as area granulometry, boundaries, skeleton, or convex hull. In addition, morphological operators can be used for image preprocessing and postprocessing, such as morphological filtering, thinning, and especially for segmentation.

## 2. Scope of This Review

In this paper, we present a review of computer-aided methods oriented to malaria parasite detection and segmentation by MM based techniques. Most of the studies have followed Di Ruberto’s work [10], which first proposed a system to evaluate parasitaemia in the blood. The system was able to detect the parasites by using an automatic thresholding and morphological operators. A morphological approach to cell segmentation, which is more efficient than the watershed algorithm [5], was proposed. Finally, the parasites’ classification was still based on morphological operators. Since then, many systems for computer aided diagnosis of malaria have been proposed. Most of them make use of MM to process and analyse malaria-infected peripheral blood cells images. The scope of this paper is to review and analyze the recent works of different researchers in the area of malaria parasite recognition using computer vision that benefits from MM. Only few reviews exist in literature about microscopic image processing for malaria parasites. However, they are not focused on MM techniques as they analyze generic computer vision systems for malaria diagnosis, as in [11,12,13]. Furthermore, newer and promising approaches, addressing for example the problem of malaria diagnosis in remote areas [11,14], or improving significantly both the detection and the classification performances [15,16,17,18], have not been considered in the previous reviews.

The rest of the paper is organised as follows. Section 3 presents a review of the considered works, according to a typical pipeline of a computer-aided image analysis process: preprocessing, segmentation, feature extraction. All of the considered works make use of morphological operators in at least one of the phases of image analysis. Section 4 contains an overall discussion about the methods and the conclusions are expressed in Section 5.

## 3. Computer Aided Diagnosis of Malaria by Using Mathematical Morphology

This section presents a review of some of the main recent studies existing in literature regarding the analysis of malaria infected blood smears using MM.

Extensive search of articles has been made in PubMed and Google Scholar search engines based on the keywords: “malaria”, “mathematical morphology”, “automated malaria diagnosis” up to October 2017. The search includes papers published in English language. The following criteria were used to evaluate the relevance and suitability of an article in our study. Firstly, every single article has been chosen if it had a JCR rank value, according to Thomson Reuters website (https://jcr.incites.thomsonreuters.com/). In that case, the work has been considered suitable for our purposes. However, JCR value was not available for all the considered articles, so that we selected the ones with an SJR rank value (available from Scimago website (http://www.scimagojr.com/)). The remaining articles, more precisely [4,14,17,19,20,21], have been considered and included because of their innovativeness or because the results were particularly interesting and promising. Furthermore, some well known books, like [5,7,9,22], were included, as well as Schulze’s Ph.D thesis [23] and Špringl’s masters thesis [24]. Thereafter, a conference paper was included if the related conference owns a SJR impact factor. Finally, the full texts of these studies were analysed and evaluated.

A typical approach of a computer-aided method usually comprises four different image processing and analysis tasks, as follows:

1. Preprocessing.
2. Segmentation.
3. Feature extraction.
4. Classification.

Since morphological techniques have been used in the first three phases, the reviewed works have been divided into the following sub-sections: preprocessing, segmentation and feature extraction. Each sub-section contains description about methods that cope with malaria parasites (MP) stained components analysis, both on thin and thick blood smears, without distinction.

**Figure 4:**
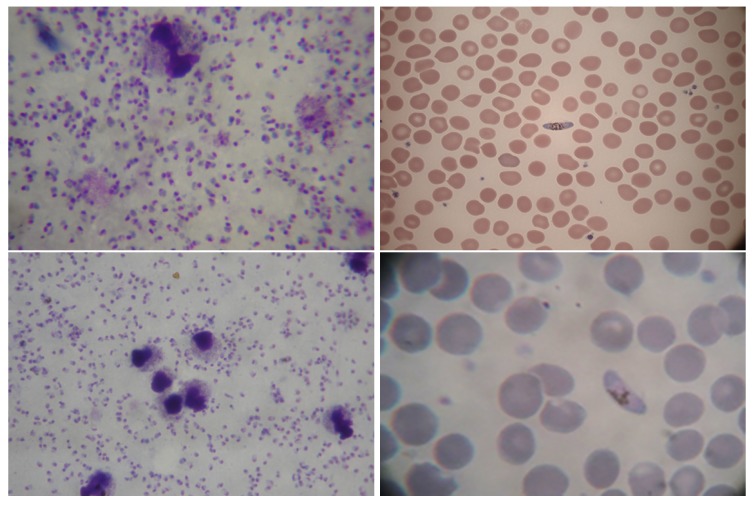
Malaria infected blood smears types. This image shows a comparison between staining colouration procedures and smears thickness. On top, left: thick smear with Giemsa stain [26], right: thin smear with Giemsa stain (courtesy of CHUV). On bottom, left: thick smear with Leishman stain [26], right: thin smear with Leishman stain (courtesy of CHUV). Dots in thick smears and rings in thin smears are P. Falciparum ring stages, while elongated erythrocytes (in images on the right) are affected from P. Falciparum in its trophozoite schizont stage. The difference between thick and thin smears is clearly evident by observing cells and parasite shapes. Thin smears typically offer a better shape representation, while thick ones contain smaller and less clear region shapes. Furthermore, Giemsa stain shows a better contrast between cells, parasites and background respect to Leishman stain.

Two main factors are generally considered if we refer to staining techniques: the type of colouration, in which Giemsa and Leishman are the most common, and the thickness of blood slide, which may be thick or thin. The majority of studies have been employed on thin blood smear images (over two-third of the total count) while only a few have used thick blood smear images. Typically, thin smears permit the identification of specific parasitic stage and quantification of malaria parasite; on the other hand, thick smears are better if the target is to perform an initial identification of malaria infection using blood pathology. Some examples are shown in Figure 4. Giemsa stained blood smear is considered in most of the analysed literatures, whereas the Leishman stain is considered in few studies. It is reported that the Leishman stain has more sensitivity for parasite detection than Giemsa [25] and is superior for visualization of red and white blood cell morphology [26]. On the contrary, Giemsa stain highlights both malaria parasites and white blood cells and, therefore, it is an additional issue to deal with. The Giemsa stain is a more costly and also time-consuming procedure than Leishman. Moreover, magnification of 100X by using an oil immersion objective is used for capturing microscopic images of thin blood smear for identification of specific parasites and their infected stage.

### 3.1. Preprocessing

In an image analysis field, especially when we refer to complex computer-aided pipelines, preprocessing methods are particularly used in order to improve the image data by suppressing unwanted noise or enhancing some image features for further processing. It is worth mentioning preprocessing methods because they are an important step regarding the image analysis field, but, for what concerns the malaria-affected blood image analysis, in our review, we particularly found methods that operate for illumination correction and noise filtering purposes. Generally speaking, digital microscopy images can be acquired in different lighting conditions, with several types of acquisition devices or from blood smears stained with various staining protocols, and, consequently, the features of similar images could differ a lot. Different techniques for illumination correction have been suggested to reduce such variation, e.g., a lot of authors work with grayscale-converted images as an illumination correction method. On the other hand, noise filtering aims to remove the noise introduced by mishandling the slides and/or the camera settings. Morphological operators have been extensively used as preprocessing for image enhancement in major studies. Erosion and dilation operations on raw smear images allow for discarding undesired patterns and help in the selection of required cells or regions of interest. Morphological operators are useful for removal of unwanted objects, holes filling, splitting, thinning and thickening. Different researchers during automated diagnosis of malaria used morphological operations in the preprocessing phase, and the most recent are listed below.

In [27], Gonzalez-Betancourt et al. proposed a system to determine markers for watershed segmentation based on the Radon transform and mathematical operators. In the first step of the process, small irrelevant structures and part of the noise are eliminated by a morphological filter, in order to ensure the preservation of the cells edges. Image smoothing is performed by a morphological erosion-reconstruction and dilation–reconstruction filter with a disk structuring element of a radius equal to 20 pixels, which is 0.274 times smaller than the average radius of the RBCs.

In this way, the influences of the size and the shape of the structures can be separated in the smoothing process. At the same time, the objects that are not eliminated remain unchanged. In addition, a morphological closing is performed with a disk structuring element having a radius smaller than half the average of the RBCs radii, in order to connect the possible (more than one) markers that can appear on a single cell.

In [28], Kareem et al. illustrated a morphological approach for blood cell identification and use the image features such as intensity, histogram, relative size and geometry for further analysis. Before the identification of blood cells, the authors propose a novel morphological filtering based on the size of RBCs for platelets and/or artifacts elimination. A dilation is performed by a concentric ring structuring element and erosion by a disk-shaped structuring element. The radius of the structuring element depends on the radius of the RBCs, so that all the components smaller than the RBCs can be removed.

The system proposed in [14] by Oliveira et al. is based on image processing, artificial intelligence techniques and an adapted face detection algorithm to identify Plasmodium parasites. The latter uses the integral image and haar-like features concepts, and weak classifiers with adaptive boosting learning. The search scope of the learning algorithm is reduced in the preprocessing step by removing the background around blood cells by means of morphological erosions both for training and for testing.

Romero-Rondon et al. in [29] presented an algorithm that uses morphological operations, the watershed method, the Hough transform and the clustering method of k-means to detect overlapped RBCs. In the preprocessing stage, white blood cells and platelets are removed before the segmentation task. During this step, some noise, the WBC cytoplasm and platelets still remain on the image.

Therefore, the small objects are removed using a morphological opening and then the image is dilated with a disk-shaped structuring element.

Reni et al. in [30] described a new algorithm for morphological filtering of the blood images as a preprocessing tool for segmentation. Conventional morphological closing on blood images removes the unwanted components but also useful information. On the contrary, the proposed method preserves the necessary information of foreground components while removing noise and artefacts.

In the method proposed in [31] by Sheikhhosseini et al., the first phase is the stained object extraction that detects candidates’ objects that can be infected by malaria parasites using intensity and colour. Before detecting the stained objects, the method firstly extracts the foreground. The foreground image is a binary image that is produced after applying morphological hole filling on such pixels that have lower intensity value than average intensity value of the green layer. After the stained objects’ extraction process, a series of morphological operations is also employed in order to eliminate small components and complete the final stained objects.

An edge-based segmentation of erythrocytes infected with malaria parasites using microscopic images is proposed by Somasekar et al. in [32]. A fuzzy C-means clustering is applied to extract infected erythrocytes, which is further processed for the final segmentation. A morphological erosion is used to erase some small noises and spots before the segmentation and holes inside the infected erythrocytes are filled using a morphological hole filling operation for the final segmentation.

In [33], Tek et al. presented a complete framework to detect and identify malaria parasites in images of Giemsa stained thin blood film specimens. In addition, the system is able to identify the infecting species and life-cycle stages. The preprocessing step of the proposed method is applied to reduce the variations in the observed size, intensity, and colour of the cells and stained objects before the detection and classification steps. The aim is to correct the non-uniform illumination in the images. The estimation is based on a morphological closing operation using a sufficiently large structuring element. The sufficiently large size for an input image is determined automatically with respect to its average cell size computed from the area granulometry distribution.

### 3.2. Segmentation of RBCs and Parasites

Segmentation is a key step in image analysis because it permits the identification and separation of the regions that compose an image, according to certain criteria of homogeneity and separation. Its main target is to divide the image into parts that have a strong correlation with objects or areas of the real world contained in the image. The commonly used segmentation methods essentially operate considering characteristics such as the brightness value, colour and reflection of the individual pixels, identifying groups of pixels that correspond to spatially connected regions. As for many problems of image processing, there is no standard solution valid in general, so different segmentation techniques can be applied, according to the characteristics of the images to process and of the objects to segment. Medical images segmentation is typically performed using two main strategies: the first level aims to separate whole cells or tissues from the background and the second one aims to separate the tissue structure in different regions or the cell in their components, as the nucleus from the cytoplasm or intracellular parasites. The latter case is commonly used in applications in which the cell class depends on the morphological characteristics of its components.

Several other authors attempted to use thresholding combined with morphological operation as a segmentation method in their computer-aided systems, and they are described as follows.

Arco et al. in [34] worked on thick blood films and proposed a method that uses an adaptive thresholding based scheme, which also allows an effective classification of pixels. This means that the election of whether a pixel belongs to the background or to the signal (parasites and white blood cells) is only established by the pixels around it, that is its neighbourhood. Then, morphological methods are applied to evaluate the area of connected components, labelling those belonging to parasites and counting their number.

Anggraini et al. [35] proposed a method for separating blood cells, parasites and other components from the background in a microscopic field of a thin blood smear. They applied several global thresholding methods and visually compared the results to qualitatively determine which technique yields the best result. The binary image was then subjected to a hole filling morphological operator and applied as a marker to label blood cells. From each identified cell (RBC and WBC), constituents of the parasite (nucleus and cytoplasm) were extracted using multiple thresholds.

Dave et al. in [15] performed image segmentation using histogram based adaptive thresholding followed by mathematical morphological operations (erosion and dilation). The detection of infected RBCs is based on an unsupervised learning technique.

The automated method proposed in [36] by Elter et al. for parasite detection and identification worked on thin blood film acquired with Giemsa stain. The authors found that the G and B channels of the RGB colour are very good features to identify objects containing chromatin in Giemsa stained blood films, being not only considered highly discriminative but also almost independent of differences in illumination and staining intensity. They transformed the colour input image into a monochrome image *I*(*x*,*y*), which highlights objects containing chromatin: $I\left(x,y\right)\;=\;arctan\frac{I_{green}\left(x,y\right)}{I_{blue}\left(x,y\right)}$. In this work, mathematical morphology has been used with a black top-hat operator to separate MP from both leukocytes and platelets, with a non-flat paraboloid structuring element with a radius of 9 and a slope of one pixel. It should be taken into account that these fixed parameters might not be suitable for images with different pixel resolutions. The black top-hat operator is followed by a thresholding operation with a fixed threshold, which, according to the authors, is reliable given the independence of the G and B channels with regard to illumination and staining intensity. However, the authors do not define the value of this fixed threshold on the publication.

Kareem et al. in [37] used the Annular Ring Ratio transform method. Before applying it, a preprocessing phase for removing platelets, parasites and other artefacts in the image has been performed. In the proposed method, the image after being converted to grayscale undergoes a morphological opening similar to closing. Unlike conventional closing (dilation followed by erosion), which uses the same structuring element, two different structuring elements are used: a concentric ring for dilation and a disk for erosion. The inner and outer diameter of the dilation ring is set to 35% and 70% of RBCs size, respectively, and the erosion disk has the same diameter. Therefore, considering that fixed manually defined parameters are used for this strategy, the results may substantially differ depending on the image resolution. This approach results in locating only the stained components in the image instead of all the cells and hence will not only speed up the operation but reduce the complexity.

Mushabe et al. [38] used morphological and statistical classification to detect malaria in blood smears by identifying and counting red blood cells and Plasmodium parasites. Morphological operations and histogram-based thresholding are used to extract RBCs and boundary curvature calculations and Delaunay triangulation are used for splitting clumped RBCs. They worked on Giemsa-stained thin blood smears.

In [39], Ross et al. proposed a method that provides a positive or negative diagnosis of malaria and differentiates parasites by species. The segmentation step relies on a six step thresholding selection strategy. It aims to identify and segment potential parasites and erythrocytes from background. Mathematical morphology has been used in several key steps of the procedure. Hole filling is used in the first step in order to fill RBCs’ binary masks obtained from a first thresholding. Afterwards, step 4 employs RBCs’ morphological reconstruction with parasites’ mask, found in step 2, for identifying infected cells. In step 5, a morphological opening filter, using a disk-shaped SE with a radius equal to the mean erythrocyte radius less the standard deviation, is applied to the grayscale, morphologically filtered, green component in order to remove any objects smaller than an erythrocyte. The morphological gradient (difference between a dilation and erosion of the image) is then calculated using a diamond-shaped SE with unity length. Finally, in step 6, the intersection of morphological gradient image and the dilated cell cluster is calculated. This image is then transformed to a binary image by thresholding any value greater than zero. A series of morphological operations, namely a closing operation, thinning, and spur-removal are then applied to generate a contour of the segmented erythrocytes. Contours are filled, and the segmented mask is again reconstructed with the valid parasite marker image to result in a segmented mask of infected cells. RBCs and parasites masks are consequently ready for the next generation step.

Savkare et al. [21] worked on thin blood films with Giemsa staining and used a global threshold and Otsu threshold [40] on grayscale enhanced image (green channel) for separating foreground from background. Hole filling has been performed on identified cells, and morphological operators have been used to identify overlapping cells. Then, a watershed transform has been applied for separating overlapped cells.

In addition, in the method proposed in [17] by Somasekar et al., the segmentation of the infected parasites is based on thresholding. It is achieved in two stages by maximizing between-class variance of an original image and consequently by an iterative threshold selection from a stage-one threshold image with suitable stopping criteria. The segmented results are postprocessed to improve the accuracy of malaria parasites detection by morphological operators (erosion and closing).

On the other hand, a lot of works have been realized by means of mathematical morphology and/or granulometry in the segmentation stages, even in combination with thresholding strategies. They are briefly analysed below.

Ahirwar et al. [41] based their approach on thresholding and granulometry. The histogram of the complemented green component has been used, and it is said to be a bimodal distribution in all the considered images. Then, both local and global thresholds are used, and the union of the two binary images is chosen as the parasite marker image. A morphological opening filter, using a disk-shaped SE with radius equal to the mean erythrocyte radius less the standard deviation, is applied to the grayscale morphologically filtered green component of the image to remove any objects smaller than an erythrocyte. The morphological gradient is then calculated using a diamond-shaped SE with unity length. The segmentation method is applied to each object in the reconstructed binary image of erythrocytes individually. Those objects that do not exceed the area of a circle with a radius equal to the mean erythrocyte radius plus the standard deviation are regarded as being single cells, and are unmodified. On the other hand, the clumped cells are segmented as follows. First, the intersection of the morphological gradient image and the dilated cell cluster is taken. This image is then transformed to a binary image by thresholding any value greater than zero. A series of morphological operations, namely a closing operation, thinning, and spur removal are then applied to generate a contour of the segmented erythrocytes. The contours are filled, and the segmented mask is again reconstructed with the valid parasite marker image to result in a segmented mask of infected cells.

Di Ruberto et al. [10] aimed to detect the parasites by means of an automatic thresholding based on a morphological approach applied to cell image segmentation, which is more accurate than the classical watershed-based algorithm. They applied grey scale granulometries based on opening with disk-shaped elements, flat and hemispherical. They used a hemispherical disk-shaped structuring element to enhance the roundness and the compactness of the red cells improving the accuracy of the classical watershed algorithm, while they have used a disk-shaped flat structuring element to separate overlapping cells. These methods make use of the red blood cell structure knowledge, which is not used in existing watershed-based algorithms.

Khan et al. in [19] presented a novel threshold selection technique used to identify erythrocytes and possible parasites present on microscopic slides that greatly benefits from morphological operations, such as granulometry and morphological reconstruction.

In [11], Rosado et al. proposed a system using supervised classification to assess the presence of malaria parasites and determine the species and life cycle stage in Giemsa-stained thin blood smears. For the RBCs segmentation, they used an adaptive thresholding approach followed by a closing morphological operation with an elliptical structuring element.

Soni et al. [42] performed segmentation of erythrocytes by using granulometry as well. The size and eccentricity of the erythrocytes are also required for the calculation of some feature values (as these can be indicative of infection). The shape of the objects (circular erythrocytes) is known a priori, but the image must be analysed to determine the size distribution of objects in the image and to find the average eccentricity of erythrocytes present. Grayscale granulometries based on opening with disk-shaped elements are then used. Non flat disk-shaped structural elements are applied to enhance the roundness and compactness of the red blood cells and flat disk-shaped structural elements applied to segment overlapping cells. The object to be segmented differs greatly in contrast to the background image. Changes in contrast can be detected by operators that calculate the gradient of an image. The gradient image can be computed and a threshold can be applied to create a binary mask containing the segmented cell. The binary gradient mask is dilated using a vertical structuring element followed by a horizontal structuring element. The cell of interest has been successfully segmented, but it is not the only object that has been found. Any objects that are connected to the border of the image can be removed.

In Tek et al. [33], the localisation of the parasites is achieved after a foreground and background segmentation step. Firstly, a rough foreground image using morphological area top-hats (using the average cell area value) is extracted. Then, from these rough foreground and background regions, two different threshold values are determined and used in morphological double thresholding of the input grey level image to produce a refined binary foreground mask. From the foreground image, the stained pixels are detected using again a thresholding approach and finally used as markers to extract the stained objects by morphological area top-hats based on the estimated average area value.

In [43], Yunda et al. proposed a method for *P. vivax* parasite detection. The segmentation phase is a combination of border and region detection that allows rejection of the image background and permits identifying each of the objects. Initially, the morphological gradient method is used to enhance the borders of previously found objects. This is followed by a threshold detection stage using the K-Median method. Furthermore, a Laplacian operator was used to discriminate the pixels that are interior or exterior in relation to the regions of the images and then erosion operation followed by two dilations were applied to delete the pixels that did not make up part of any object. In the end, Absence of Gradients and Nernstian Equilibrium Stripping (AGNES) and K-Median techniques were applied to assign the remaining number of pixels to each region, using the image regions previously identified as objects and background as the starting point.

Several authors used marker controlled watershed [5] with morphological approach, as described in the following.

Das et al. in [12,22,44,45] segmented erythrocytes as aforesaid and then morphological operators are used to eliminate unwanted cells like leukocytes and platelets. To conclude, overlapping erythrocytes are segmented by using a marker controlled watershed segmentation technique.

In [16], Devi et al. proposed a computer assisted system for quantification of erythrocytes in microscopic images of thin blood smears. The performance of the system in classifying the isolated and clump erythrocytes by geometric features is evaluated for the different classifiers. The clump erythrocytes are segmented using marker controlled watershed with h-minima as internal marker.

In [46], Dey et al. presented an automatic system for segmenting platelets, useful for identifying disease as malaria, using a colour based segmentation and mathematical morphology (opening operations with a disk element of radius 2).

In the study presented in [47] by Diaz et al. for quantification and classification of erythrocytes in stained thin blood films infected with *Plasmodium Falciparum*, the authors used connected morphological operators in the segmentation step. The RBCs are detected as follows: firstly, a pixel classification allowed for labeling each image pixel as either background or foreground, based on its colour features. Afterwards, an inclusion-tree structure is used to represent the hierarchical object relations between background and foreground so that a filtering process allows for removing irrelevant structures such as artefacts generated at the staining or digitization processes.

Khan et al. [19], among other experimentations, used it in order to try to separate overlapping cells because, according to their statements, watershed transform can separate touching cells, but it is not sufficient for overlapping cells.

In the algorithm described by Romero-Rondon et al. in [29], the detection of overlapped RBCs is still based on marker-controlled watershed transform. To define the suitable markers in watershed transform, they used three different approaches, based on a morphological erosion operation, on Hough transform and on a clustering method of K-means.

Savkare et al. in [48] segmented cells using K-mean clustering and global threshold. Overlapping cells are separated using a Sobel edge detector and watershed transform. Watershed transform is applied on each cluster separately. Over-segmentation is minimized by a series of morphological operations, like erosion and dilation utilizing disk-shaped structuring elements.

In [20], an approach to detect red blood cells with consecutive classification into parasite infected and normal cells for further estimation of parasitemia is proposed. For separation of overlapping cells, watershed transform is applied on a distance transform of binary mask of cells having a larger area.

In [24], Špringl performed red blood cell segmentation by using marker-controlled watershed transformation based on the image gradient. Markers are computed as a combination of the binary mask of the red blood cells and centres of the cells that are computed using a similar algorithm that was utilized for the evaluation of the average cell radius. The binary mask is obtained by thresholding the grayscale image with an automatically estimated threshold using the Otsu method [40].

In [18], Sulistyawati et al. combined morphological operations (erosion, dilation, opening and closing) and blob analysis to segment and identify malaria parasites with a high degree of accuracy.

Tek et al. in [49] proposed a classifier-based method for the segmentation stage, which relies on a Bayesian pixel classifier to distinguish between stained and non-stained pixels. In particular, they used a non-parametric method based on histograms in order to produce the probability density functions of stained and non-stained classes. Stained pixels can belong to other components such as WBCs, platelets or artefacts, in addition to the parasites, and so the detection procedure requires a further classification to distinguish among parasite and non-parasite pixels. However, the stained pixels have to be represented as connected sets, representing stained objects, to extract features for the classifier. Furthermore, top-hat extraction and infinite reconstruction were applied to find the regions that include the objects.

To sum up, the analysed systems use mathematical morphology methods during or immediately following the segmentation step for the following purposes:

- size evaluation of regions obtained from segmentation
- image cleaning and artefacts removal
- RBCs and parasites segmentation or separation
- improving parasites detection
- minimization of watershed over-segmentation.

### 3.3. Feature Extraction

Feature extraction has the aim of reducing the computational complexity of the subsequent process and facilitating a reliable and accurate recognition for unknown novel data, considering that the input data to an algorithm could be too large to be processed and it could be redundant (e.g., repetitiveness of pixels patterns in an image). Moreover, the in-depth understanding of the domain-specific knowledge gained by human experts on the problem being addressed can be of extreme importance for the design of a reliable and effective feature extraction engine [50]. It starts from determining a subset of the initial features, and this procedure is called feature selection. The selected features are expected to contain the relevant information from the input data, so that the desired task can be performed by using this reduced representation instead of the complete initial data. Malaria parasite infection causes micro structural changes in erythrocytes. The microscopic features of the RBCs are usually specific to morphology, intensity and texture. They may also represent the differences that occur among healthy and unhealthy cells. Most of the studies have reported both textural and geometric features for describing malaria infection stages [12]. Generally speaking, features may be distinguished according to the following characteristics: morphological features and textural and intensity features.

It is a well known mathematical morphology approach to compute a size distribution of grains in binary images, using a series of morphological opening operations. It is the basis for the characterization of the concept of size. Some authors used area granulometry for preprocessing purposes in malaria characterization [33], even though it is certainly effective for extracting cell size features’ information [24,49,51]. In [33], local area granulometry combined with colour histogram are used as features. The area granulometry feature is calculated locally on the binary mask of the stained objects, for the RGB channels, and then concatenated. Morphological features are also used in [44] (opening, closing) and in [10] (skeleton) to classify parasites.

## 4. Discussion

In the review, we have only considered the methods that employed mathematical morphology in at least one step of the pipelines, and it has been structured by considering the following information: preprocessing, segmentation, features extraction. Most of the studies are based on *P. vivax* and/or *P. falciparum* characterization. With regards to the approaches shown and related results, it is clear that malaria parasite detection and segmentation techniques in microscopic images need further experiments and improvements. In general, the analysed works have been tested with a limited number of images and the datasets are not publicly available; therefore, a comparison between different approaches is very difficult. Despite promising results reported during the past few years, the great majority of the computer-aided methods found on the literature for malaria diagnosis are based on images acquired under well controlled conditions and with proper microscopic equipment. However, one should take into account that 80% of malaria cases occur in Africa, where this type of equipment is scarce or even nonexistent in common healthcare facilities [11]. Moreover, this review showed that *P. falciparum* is the most analysed if we refer to segmentation and detection, considering that it is the most widespread among malaria parasite types. The majority of the works used thin blood smear. It is typically used for identification of malaria infected stages, types of parasitic infection and percentage of parasitemia, while thick blood smear is used for identification and quantification of malaria parasite count against leukocyte count per microliter of blood.

The preprocessing phase is typically taken on with filters and the most used in the analysed works is certainly the median filter, which permits preserving sharp edges. Apart from the classic histogram equalization and contrast stretching techniques, other filters have been employed, e.g., geometric mean filter to remove Gaussian noise preserving edges, Laplacian filter in order to find edges, and so on. A median filter has been found to be effective for reducing impulse noises from the microscopic images, even though recent studies have shown that geometric mean filter provides better performance than the median filter [12,45]. However, morphological operators have been greatly used with successful performances, imposing themselves as powerful alternatives to more common and used techniques for image enhancement and noise filtering [10,14,24,27,28,29,30,31,32,33,37,38,39,51].

Malaria parasites may be discriminated according to two different strategies: by segmenting the whole erythrocyte from the blood smear image on the basis of which malaria infection is detected, or by segmenting chromatin dot or parasite infection regions for characterizing parasite infection stages based on some extracted target features. In general, a thresholding-based approach is still widely used for segmentation purposes. In particular, a lot of authors affirm that Otsu thresholding suffers from limitations when textural variation is high, while histogram thresholding can not deal sufficiently well in identifying valley regions in the case of unimodal histograms. However, such a simple and fast approach can greatly benefit from mathematical morphology as recent studies demonstrate [10,11,17,21,33,34,35,36,37,38,39,48].

Another often used segmentation approach is clearly the watershed transform. The classic watershed approach is reported to produce over segmentation results [21], whereas the marker controlled approach does not suffer from this issue, and it is reported to be very effective for overlapping cells’ segmentation even though some authors affirm that it may fail to segment highly overlapped cells [12,16,19,20,22,24,29,44,45,48].

Other authors [10,19,33,38,39] employed granulometry and stated that it is very effective to segment cells with regular size.

The analysed works performed classification phase for different purposes. The majority of them aimed to distinguish among two classes only, malaria infected and noninfected RBCs, or to detect and count parasites in a malaria blood image [4,10,12,14,15,17,18,20,21,25,28,32,34,35,36,38,44,49,51].

More complex classification strategies aimed to classify parasites into different classes, i.e., different human parasites species [19,22,33,45], and/or different parasites life stages [10,22,33,35,45,47].

A summary of analysed methods is shown in Table 1. For each approach, we report the processing phase where MM is used (preprocessing and/or segmentation), which operators are applied, which type of morphology (grayscale or binary), and shape and size of the used SE, if described. We detail the kind of classification, i.e., if a method addresses the detection problem (infected and non infected) only, if it faces the labelling of different parasites (*P. falciparum*, *P. ovale*, *P. malariae* or *P. vivax*) and, in some cases, the different stages of life (ring, trophozoite, schizont, gametocyte). Finally, the measures used for evaluating the system performance are reported, if present.

## 5. Conclusions

This work reviewed several computational microscopic imaging techniques oriented to a mathematical morphology approach, proposed in literature for malaria parasite detection and segmentation in blood smear microscopic images.

The computer vision methodologies reported in the literature are based on light microscopic images of human peripheral blood smears for computer-aided detection of malaria parasites and their different life stages. Image preprocessing, segmentation of erythrocytes and parasites, malaria parasite feature extraction, and malaria detection techniques have been discussed here.

It is worth noticing that cell colour and the colour contrast between cells and background can vary so often according to the different, existing staining techniques, thickness of smear, microscope illumination and microscope’s image acquisition procedure, as shown in Figure 1. A standardization of the procedure should be really useful to avoid superfluous differences in similar images’ features and to have fair comparisons among the several proposed methods. Moreover, independence of sample cannot be overlooked. Usually, any pattern recognition system is evaluated by performing tests using a real set of samples. In the case of diagnosis, this is a key aspect. Any system for diagnosis should be tested on different images, acquired by different sources, and different specimens. This is crucial to guarantee the diagnostic capability and usefulness of a computer vision system. The main efforts towards the realization of a fully automatic blood cells segmentation and classification system cannot leave these aspects out.

Mathematical morphology techniques have been widely used for image processing purposes. Among the application fields, it has been applied for fingerprint feature extraction, recognition of handwritten digits, license plate detection, border extraction, denoising using morphological filters, text extraction and so on. Apart from these kinds of fields, mathematical morphology has been employed successfully in biomedical image analysis, especially in preprocessing and segmentation techniques.

Morphological cell analysis is used to face off abnormality identification and classification, early cancer detection. It has been integrated in new methods for biomedical applications, such as automatic segmentation and analysis of histological tumour sections, boundary detection of cervical cell nuclei considering overlapping and clustering, the granules segmentation and spatial distribution analysis, morphological characteristics analysis of specific biomedical cells, understanding the chemotactic response and drug influences, or identifying cell morphogenesis in different cell cycle progression. Morphological feature quantification for grading cancerous or precancerous cells is especially widely researched in the literature, such as nuclei segmentation based on marker-controlled watershed transform and snake model for hepatocellular carcinoma feature extraction and classification, which is important for prognosis and treatment planning, nuclei feature quantification for cancer cell cycle analysis, and using feature extraction including image morphological analysis, wavelet analysis, and texture analysis for automated classification of renal cells [52].

Moreover, nonlinear filtering has become increasingly important in many image processing applications. Initially, the attraction to nonlinear filters was mostly limited to the impulse-removing and edge-preserving qualities of the median filter. However, as the number and sophistication of nonlinear filters have increased, so has the variety of applications for these filters. The shape-based methods of mathematical morphology, in particular, are now used in a wide variety of medical applications, including electrocardiography, ultrasound imaging, radiology, and histological image analysis [23].

Furthermore, microscopic image analysis and, in particular, malaria detection and classification can greatly benefit from the use of mathematical morphology. The interest in this approach to image processing ad analysis is proved by the increasing number of works proposing methods for malaria image analysis based on mathematical morphology techniques. Mainly, morphological techniques have been used in the preprocessing and segmentation phases, both in grayscale and in binary. The most used operators have been dilation, erosion, opening and closing in order to improve the quality of the image in both of the phases, marker controlled watershed and granulometry to segment and detect RBCs and parasites. No proposed system for malaria diagnosis is based on colour morphological operations. Since such techniques have been already applied for shape analysis, border detection through morphological gradient, segmentation, and texture analysis, a morphological approach using colour information could be extremely useful in blood smear processing and analysis.

In the end, it is worth considering that the development of new mobility-aware microscopic devices (and ideally low cost) is an area that can greatly improve the chances of the successful deployment of computer vision CAD solutions for malaria diagnosis in the field. The mobile phone is currently Africa’s most important digital technology, and is boosting African health as it emerges as a platform for diagnosis and treatment. Considering the recent significant improvements of the new generation of mobile devices in terms of image acquisition and processing power, if a reliable automatic diagnostic performance is ensured through the usage of those devices, one would dramatically reduce the effort in the exhaustive and time-consuming activity of microscopic examination. Moreover, the lack of highly trained microscopists on malaria diagnosis in rural areas could then be complemented by a significantly less specialized technician that knows how to operate the system and prepare blood smears. The usage of mobile devices in the system architecture can also bring significant improvements in terms of portability and data transmission, like the systems proposed by [11,14]. Finally, malaria diagnosis might be just one element of a suite of diagnostic software tests running on this type of system. Several other tests could simultaneously be carried out using the same images, for instance cell counting or detection of other hemoparasites, like microfilaria or trypanosoma [53].

## Figures and Tables

**Table 1:** Summary of analysed methods: morphological operations used in the main phases of analysis, type of MM (gray or binary)/type and size of SE (if reported), kind of classification and performance measures (Sensitivity, Specificity, Accuracy, if reported).

Authors	Preprocessing	Segmentation	MM/SE	Classification	Performance
Ahirwar et al., 2012	-	thresholding + granulometry, opening, morphological gradient, dilation, closing, thinning, spur removal	gray, bin/disk of size depending on RBCs, diamond of size = 1	five (*P.falciparum*, *P.vivax*, *P.ovale*, *P.malariae* infected, and noninfected)	-
Anggraini et al., 2011	-	thresholding + hole filling	bin	two (*P.falciparum* infected and noninfected) + two life-cycle-stages	SE = 93% SP = 99%
Arco et al., 2014	-	adaptive thresholding + hole filling, closing, regional minima	gray, bin/disk	two (infected and noninfected)	Acc = 96.46%
Das et al., 2011	-	marker controlled watershed + opening, closing	gray, bin	two (infected and noninfected)	Acc = 88.77%
Das et al., 2013	-	marker controlled watershed	gray	three (*P.falciparum*, *P.vivax* infected and noninfected) + three life-cycle-stages per species	Acc = 84%
Das et al., 2014	-	marker controlled watershed	gray	three (*P.falciparum*, *P.vivax* infected and noninfected) + three life-cycle-stages per species	SE = 99.72% SP = 84.39%
Dave et al., 2017	-	adaptive thresholding + erosion, dilation	bin	two (infected and noninfected)	Acc = 97.83% thin films, Acc = 89.88% thick films
Devi et al., 2017	-	marker controlled watershed	gray	two (infected and noninfected)	Acc = 98.02%
Diaz et al., 2009	-	inclusion tree	gray	two (*P.falciparum* infected and noninfected) + three life-cycle-stages	SE = 94% SP = 99.7% for detection, SE = 78.8% SP = 91.2% for life-stages
Di Ruberto et al., 2002	area closing, opening	thresholding + granulometry, watershed transform + skeleton	gray, bin/disk, flat and nonflat, with size depending on RBCs	two (*P.falciparum* infected and noninfected) + three life-cycle-stages	-
Elter et al., 2011	-	thresholding + black top-hat, dilation	gray/disk, nonflat with size = 9	two (infected and noninfected)	SE = 97%
Gonzalez-Betancourt et al., 2016	morphological filter, erosion-reconstruction, dilation–reconstruction, closing	watershed transform	gray/disk with size depending on RBCs	-	-
Kareem et al., 2011, 2012	dilation, erosion	-	gray/concentric ring, disk with size depending on RBCs	two (infected and noninfected)	Acc = 88% SE = 90% SP = 86%
Khan et al., 2011	area closing	thresholding + granulometry, opening, morphological reconstruction, gradient, dilation	gray	five (*P.falciparum*, *P.vivax*, *P.ovale*, *P.malariae* infected, and noninfected)	Acc = 81% SE = 85.5%
Malihi et al., 2013	closing	area granulometry	gray/disk with size depending on RBCs	two (infected and noninfected)	Acc = 91% SE = 80% SP = 95.5%
Mushabe et al., 2013	closing	thresholding + granulometry, dilation, erosion	gray, bin/disk	two (infected and noninfected)	SE = 98.5 SP = 97.2%
Oliveira et al., 2017	erosion	-	gray, bin	two (infected and noninfected)	Acc = 91%
Reni et al., 2015	new morphological filtering	-	gray/anular ring, disk with size depending on RBCs	-	-
Romero-Rondon et al., 2016	dilation, opening	marker controlled watershed, erosion	gray, bin/disk with size depending on RBCs	-	-
Rosado et al., 2017	-	adaptive thresholding + closing	bin/elliptical with size = 3	four (*P.falciparum*, *P.ovale*, *P.malariae* infected, and noninfected) + three life-cycle-stages for species	SE = 73.9–96.2% SP = 92.6–99.3%
Ross et al., 2006	area closing	thresholding + granulometry, opening, reconstruction, morphological gradient, closing, thinning	gray, bin/disk with size = 6 and depending on RBCs, diamond with size = 1	five (*P.falciparum*, *P.vivax*, *P.ovale*, *P.malariae* infected, and noninfected)	SE = 85% for detection, Acc = 73% for classification
Savkare et al., 2011a	-	thresholding + hole filling, watershed transform	bin	two (infected and noninfected)	-
Savkare et al., 2011b	-	thresholding + hole filling, watershed transform	bin	two (infected and noninfected)	SE = 93.12% SP = 93.17%
Savkare et al., 2015	-	thresholding + watershed transform, erosion, dilation	bin/disk with size = 2	two (infected and noninfected)	Acc = 95.5%
Sheikhhosseini et al., 2013	hole filling	thresholding + hole filling, opening	bin	two (infected and noninfected)	Acc = 97.25% SE = 82.21% SP = 98.02%
Somasekar et al., 2015	erosion	fuzzy C-means clustering + erosion, hole filling	bin/square with size = 3	two (infected and noninfected)	SE = 98% SP = 93.3%
Somasekar et al., 2017	-	thresholding + erosion, closing, hole filling	bin/square with size = 3	two (infected and noninfected)	average DSC = 0.8
Soni et al., 2011	-	thresholding + granulometry, morphological gradient, dilation	gray, bin/disk, flat and nonflat, vertical, horizontal	five (*P.falciparum*, *P.vivax*, *P.ovale*, *P.malariae* infected, and noninfected)	SE = 98% for detection
Špringl, 2009	closing	thresholding + marker controlled watershed transform, hole filling, dilation, opening, erosion	gray, bin/disk with size depending on RBCs	two (infected and noninfected)	AUC = 0.98
Sulistyawati et al., 2015	-	blob analysis + erosion, dilation, opening, closing, hole filling	bin	two (infected and noninfected)	Acc = 99.39%
Tek et al., 2006	-	top-hat, infinite reconstruction, area granulometry	gray, bin	two (infected and noninfected)	SE = 74% SP = 98%
Tek et al., 2010	closing, granulometry	thresholding + granulometry, area top-hat, closing, area granulometry	gray/disk with size depending on RBCs	five (*P.falciparum*, *P.vivax*, *P.ovale*, *P.malariae* infected, and noninfected) + four life-cycle-stages for species	SE = 72% SP = 98%
Yunda et al., 2012	-	thresholding + morphological gradient, erosion, dilation	gray	three (*P.falciparum*, *P.vivax* infected, and noninfected) + two life-cycle-stages for *P.falciparum*	SE = 77.19%

## Abbreviations

The following abbreviations are used in this manuscript:

CBC	Complete Blood Count
SJR	Scientific Journal Rank
JCR	Journal Citation Reports
WBC	White Blood Cell
RBC	Red Blood Cell
MM	Mathematical Morphology
MP	Malaria Parasite
SE	Structuring Element
CHUV	Centre Hospitalier Universitaire Vaudois
